# Monitoring state of charge and volume expansion in lithium-ion batteries: an approach using surface mounted thin-film graphene sensors†

**DOI:** 10.1039/d2ra07572e

**Published:** 2023-03-01

**Authors:** Gerard Bree, Hongqing Hao, Zlatka Stoeva, Chee Tong John Low

**Affiliations:** a WMG, Electrochemical Engineering Group, Energy Innovation Centre, University of Warwick Coventry CV4 7AL UK C.T.J.Low@warwick.ac.uk; b DZP Technologies Limited, Future Business Centre Kings Hedges Road Cambridge CB4 2HY UK zlatka.stoeva@dzptechnologies.com

## Abstract

Accurate monitoring of battery cell state of charge (SoC) and state of health (SoH) is vital to the safe and effective operation of rechargeable battery systems such as those in electric vehicles yet remains a challenge while the system is in use. A new surface-mounted sensor enabling simple and rapid monitoring of lithium-ion battery cell SoC and SoH is demonstrated. Small changes in cell volume brought about by the expansion and contraction of electrode materials during charge and discharge are detected through monitoring the changes in electrical resistance of a graphene film in the sensor. The relationship between sensor resistance and cell SoC/voltage was extracted, enabling rapid SoC determination without interruption to cell operation. The sensor was also capable of detecting early indications of irreversible cell expansion due to common cell failure modes, enabling mitigating steps to be taken to avoid catastrophic cell failure.

## Abstract 2

A new graphene percolation-based resistance strain gauge can measure state-of-charge and state-of-health of lithium-ion batteries with high sensitivity.

## Graphical abstract

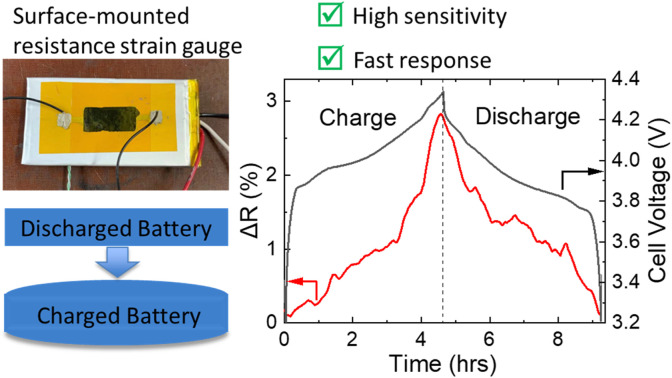

## 1 Introduction

The use of electric vehicles (EVs, both full-electric and hybrid) utilising lithium-ion batteries has seen a rapid increase in recent years. Accurate monitoring of battery state-of-charge (SoC) and state-of-health (SoH) in an EV is crucial for determination of vehicle range (functioning similar to a fuel gauge in conventional vehicles), as well as monitoring and maintaining the overall health of the battery system, yet remains elusive.^1^ This role is typically provided by the battery management system (BMS), which utilises simple current, voltage & temperature measurements to monitor SoC and SoH on a pack or module level. Popular EV models utilise cells organised into modules which are controlled by a central BMS. For instance, the Tesla Model S contains 7140 × 18 650 cells (arranged in 16 modules of 74 parallel and 6 series cells),^2^ and the BMS monitors battery voltage and temperature, and protects against overvoltage.^3^ The Nissan Leaf contains a 30 kW h battery pack consisting of 192 pouch cells arranged in 8-cell modules,^4^ and monitors SoC through open circuit voltage (OCV) and charge-counting (CC) methods.^5^ This modular BMS design has a limited scope of available data, and thus the displayed range value is a rough estimate at best. Furthermore, the module-level approach means it cannot respond to individual cell failures and thus the performance of the entire system is often limited by the weakest cells.

Recently, the concept of “smart” battery monitoring has gained traction.^6^ Within such a system, battery monitoring is performed at the cell-level by individual integrated cell BMSs, enabling greater control of cell balancing and reconfiguration.^7,8^ This approach effectively increases the performance of a battery system, enabling greater lifetimes and reducing the chance of full system failure. However, within such systems, there remains a need for more accurate determination of cell SoC and SOH.

Accurate SoC monitoring at the cell level is challenging,^9^ and several methods have been proposed, each with advantages and drawbacks. The most simple and widespread is OCV measurement. This technique relies on the principle of a distinct relationship between cell OCV and SoC, and is therefore only useful for battery chemistries in which a clear relationship exists (*e.g.* LiNi_1−*x*−*y*_Mn_*x*_Co_*y*_O_2_, NMC) and not for those in which cell voltage is relatively static over the range of SoC (*e.g.* LiFePO_4_, LFP). Furthermore, an accurate OCV measurement is only possible while the battery is not in use, limiting usefulness in EVs. A second common method, known as “charge-counting”, involves the precise measurement and logging of battery current throughout its lifetime to predict SoC. In this case, the initial SoC must be known, and small errors in current measurement accrued over the battery lifetime will lead to significant errors in the calculated SoC. Further methods utilise complex modelling combined with impedance data, requiring a heavy computational load.^10^

**Fig. 1:**
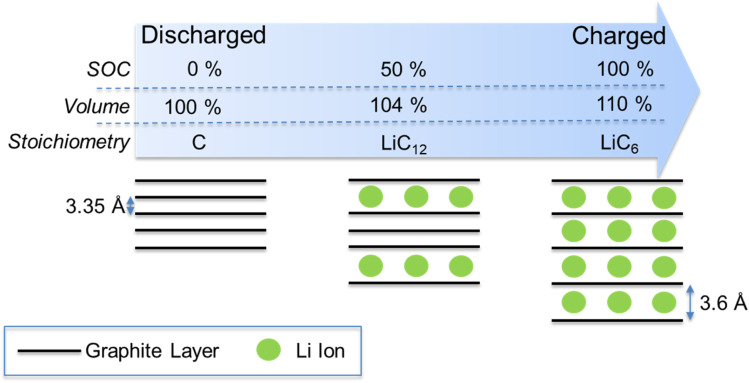
Graphic showing the structural changes and associated volumetric expansion in LIB graphite anodes during the charge (lithiation) process. The reverse occurs during discharge (delithiation).

One promising method for monitoring of cell SoC is through detection of cell dimensional changes. Lithium-ion cells undergo significant volumetric expansion and contraction during charge and discharge respectively.^11^ During cell charging, lithium ions are intercalated into the graphite anode host causing an increase in the interplane distance (from 3.35 Å to 3.6 Å), bringing about a total volume expansion of approx. 10% (Fig. 1).^12^ Since the graphite anode typically represents 35% of total cell volume,^11,13^ this corresponds to a cell-level volume expansion of approx. 3.5%. Cathode materials tend to undergo smaller volume expansions during lithiation (*e.g.* 3.4% for NMC^14^). This differential in electrode expansion brings about an overall cell volume expansion (during charge) and contraction (during discharge). Thus, accurate monitoring of this volume expansion can provide useful information on cell SoC.

In addition to the reversible volume changes observed during normal charge–discharge operation, irreversible changes associated with cell degradation often occur. These irreversible changes are brought about by gas generation, Li plating or the build-up of side-reaction products such as in the solid-electrolyte interphase (SEI).^10^ These processes (and hence the expansion) can be gradual as the cell slowly decays over its lifetime, but can also occur more rapidly during catastrophic cell failures events. Therefore, monitoring of cell expansion can provide useful information on its SOH, and crucially can enable mitigation steps to be taken by the BMS to avoid individual cell failure and increase system lifetime. Volume expansion related to increased cell temperature is also useful to monitor due to its effect on system mechanical integrity and safety.^15^

Several methods, such as dilatometry and the use of fibre bragg gratings, have been proposed to measure battery cell dimensional changes, (full details of the techniques are shown in Table S1†). These techniques often involve complex, costly, and large equipment unsuitable for incorporating into commercial EV battery systems and are thus limited to laboratory scale investigations. Given the large number of cells in a typical EV, a practical method for individual cell monitoring must have a small gravimetric and volumetric footprint, so as not to significantly reduce system energy density. Resistance strain gauges, whereby cell deformation is monitored by a change in electrical resistance of a surface mounted gauge, can fulfil this requirement and provide information on cell volumetric change with a high degree of accuracy.^16–19^ Existing commercially-available gauges rely on constructing long, complex electrical pathways (usually composed of a copper-nickel alloy^20^) to generate sufficient signal.

In this study, we assess the performance of a percolative sensor based on a simple thin film of graphene hosted in a polymer matrix as a resistance strain gauge to detect small volume changes in commercial lithium-ion battery cells. This new type of sensor represents a significant enhancement over traditional gauges whereby the exploitation of the percolative conduction mechanism within the film enables a high level of sensitivity without the need for complex sensor pathway designs, allowing for thinner, lighter, and more cost-effective monitoring of cell deformation. This opens the door to a truly “smart” BMS with cell-level monitoring, enhancing the reliability, safety, and lifetime of EVs.

## 2 Experimental

### 2.1 Manufacture of sensors

**Fig. 2:**
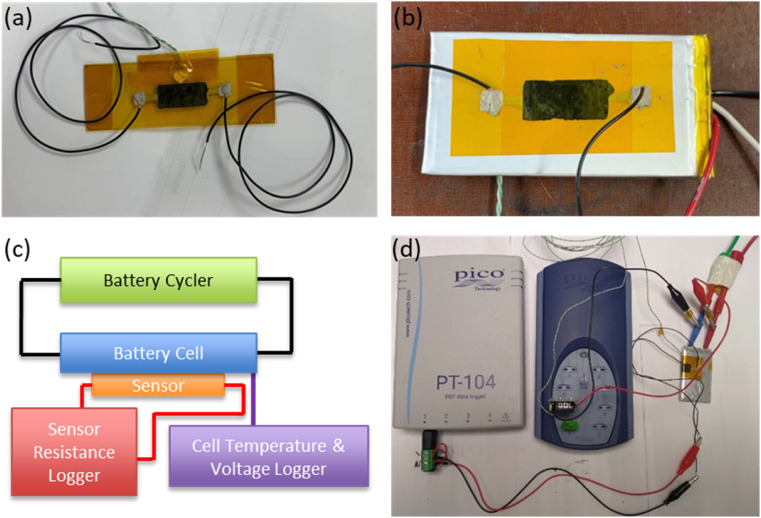
Photograph of graphene sensor (a) as produced, (b) mounted on pouch cell. (c) Schematic and (d) photograph of typical measurement setup.

The sensors were produced by bar-coating an aqueous graphene ink (product G0240 from DZP Technologies Ltd) on a polyimide flexible film of thickness 60 μm. The area of the sensing coating was 10 × 20 mm. To ensure reliable electrical connections, silver pad electrodes were printed on both sides of the sensing element, and wires were attached to the pads using a conductive adhesive (silver-filled epoxy gel, MG Chemicals). All components of the sensor assembly were dried at 120 °C for one hour to ensure that all moisture from the inks had been removed. The graphene sensing element was then laminated with another polyimide layer. In this way, the film was protected from mechanical damage and isolated from moisture and other gases in the environment which could affect sensor operation. The substrate polyimide film had an adhesive silicone backing which allowed the printed sensors to be easily attached to the surface of the battery cells as stickers. An as produced sensor is shown in Fig. 2a.

### 2.2 Mounting of sensors

The battery cell types examined in this study were pouch cells (VARTA LPP 423566 BE, 1.16 A h) and cylindrical cells (Samsung ICR18650-26J M, 2.6 A h). In this way, the application of the sensor to the most popular cell types (including both soft and hard casings) was examined. Both cell types utilised the common NMC‖Graphite chemistry (full cell characteristics are shown in Table S2†). When attaching the sensors to cell surfaces, care was taken to apply the sticker “flush” to the surface, avoiding the creation of any air bubbles. A sensor mounted on a pouch cell is shown in Fig. 2b. To monitor cell temperature, a thermocouple was placed on the cell adjacent to (but not touching) the sensor, and a thermally conductive paste (RS components) was used to provide adhesion and good thermal continuity between cell and thermocouple. The electrical resistance of the sensors was monitored and logged using a PT-104 resistance data logger (Picotech), while cell temperature and voltage were monitored using a TC-08 data logger (Picotech). Fig. 2c shows a schematic, and Fig. 2d a photograph, of the experimental setup.

### 2.3 Electrochemical testing

A range of electrochemical testing was carried out on the cells with mounted sensors, both to investigate the efficacy of the sensor in detecting volume expansion, and to simulate typical use cases. For tests in which the cells were cycled within their rated conditions, they were placed in a temperature-controlled chamber at 25 °C connected to a Biologic VMP3 potentiostat (coupled to a 5 A booster), controlled by EC-lab control software. For abuse testing (overcharge, outside rated conditions), the cells were placed in an isolated “abuse chamber” at 25 °C and cycled using a Maccor 4200 cycler controlled by MacTest 32 software. Sensor resistance, cell temperature, cell voltage and current were logged throughout.

## 3 Results & discussion

### 3.1 Principle of sensor operation

Resistance strain gauges operate based on a measurable change in electrical resistance as a response to mechanical strain. The magnitude of the response to the strain, and hence the sensitivity of the gauge, is expressed by its gauge factor (GF):1
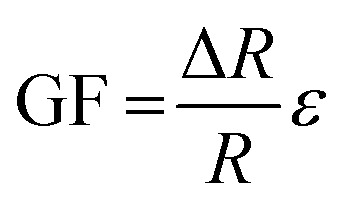
where Δ*R* is the observed change in resistance, *R* is the resistance, and *ε* is the strain (volume expansion). The graphene sensors in this work are percolative sensors in which the resistance changes due to an evolution in the structure of the conductive percolation network of graphene particles. The principle of operation of graphene percolative sensors was described in detail previously,^21^ in which it was demonstrated that this type of sensor can exhibit gauge factors (∼15) significantly exceeding those of the conventional metal strain gauges (2–5). Percolation theory proposes that the electrical characteristics of a composite material consisting of conductive particles held within an insulating matrix depend heavily on the particle concentration. Below a certain particle concentration (the percolation threshold), the conductive particles are isolated from one another and thus the composite acts as an insulator. As the percolation threshold is reached, electron transport between neighbouring particles through tunnelling becomes possible, and the conductivity increases rapidly. The relationship between composite conductivity and particle concentration above the percolation threshold can be expressed as:^22^

**Fig. 3:**
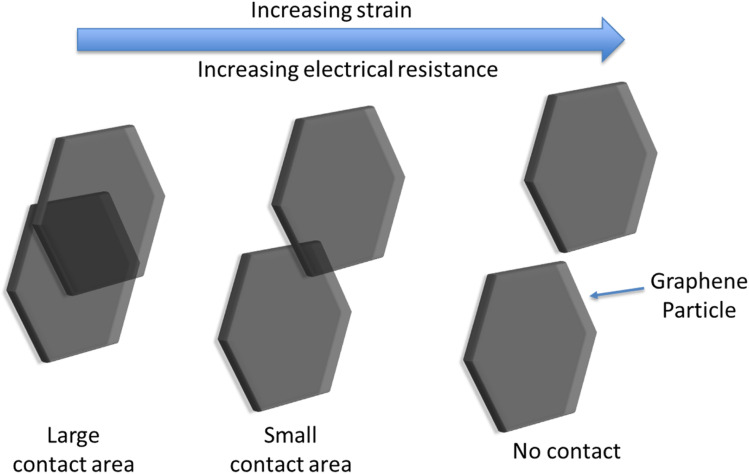
Schematic showing the effect of increasing strain on electrical conduction pathways within the sensor, demonstrating the origin of the high sensitivity.

2 *σ* = *σ*_e_(*ϕ* − *ϕ*_c_)^s^  *ϕ* > *ϕ*_c_

where *σ* is the conductivity of the bulk composite material, *σ*_e_ is the conductivity of the particles, *φ* is the particle volume fraction, *φ*_c_ is the particle volume fraction at the percolation threshold, and the exponent *s* is a constant with typical values in the range 1–2. The high sensitivity of the graphene percolative sensors can be explained by this power law dependence of the electrical conductivity on graphene particle density through the percolation threshold. During an expansion strain phase, neighbouring graphene particles move and reduce their mutual contact area (Fig. 3), and thus an increase in film resistance is observed. Furthermore, large changes in resistance (*i.e.* high sensitivity) are observed when electrical contact between particles is broken. The use of graphene is advantageous here, as the weak interparticle bonding enables neighbouring particles to slide over each other, while the high conductivity and aspect ratio of graphene enables a low percolation threshold.^23^ A further advantage of the percolation-based gauge is the ability to control the GF through simple modification of the graphene layer deposition process, enabling solutions tailored to the application *i.e.* high GF for low-strain applications.^21^

In contrast, electrical conductivity in conventional metal gauges exhibits linear dependence on the geometrical changes which take place during strain. Table 1 shows a selection of commercial-available resistance strain gauges. Given the linear dependency, these devices rely on complex design patterns to create long electrical path lengths to generate sufficient gauge factors, with limited flexibility in size and shape. This is in stark contrast to the graphene percolative sensors, whereby the simple coating process offers a low-cost of manufacture and unlimited design flexibility. In this work, we take advantage of this advantageous new sensor type, and demonstrate that the high level of sensitivity can be utilised to effectively measure battery SOC and SOH.

**Table 1:** Characteristics of selected commercially available resistance strain gauges

Manufacturer	Size	Image	Weblink
Vishay Precision Group	5.8 × 3.0 mm	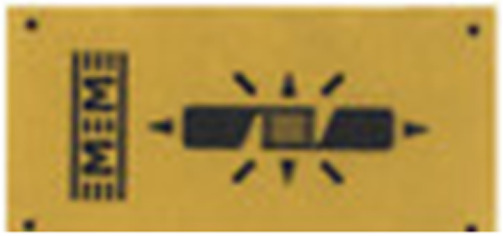	https://micro-measurements.com/pca/detail/015dja
LORD Microstrain	40 × 40 mm	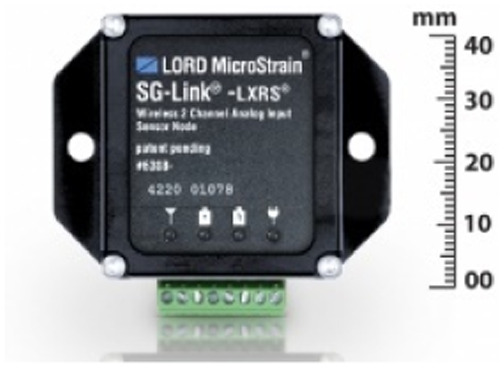	https://www.microstrain.com/all-products/strain-gauge
MFL Strain Gauges	4.4 × 2.4 mm	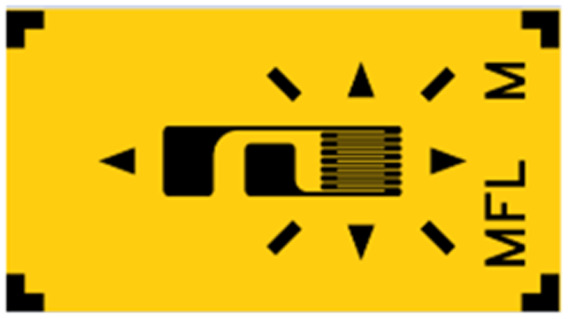	https://www.mflstraingauges.com/strain-gauges/linear/l1m-120-xx-y.html
PiexoMetrics	Length = 0.46 mm	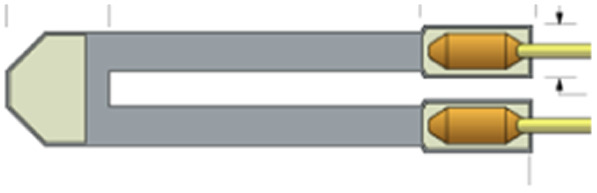	https://www.microninstruments.com/products?ProductType=1
Hottinger, Brüel & Kjær	2.0 × 1.2 mm	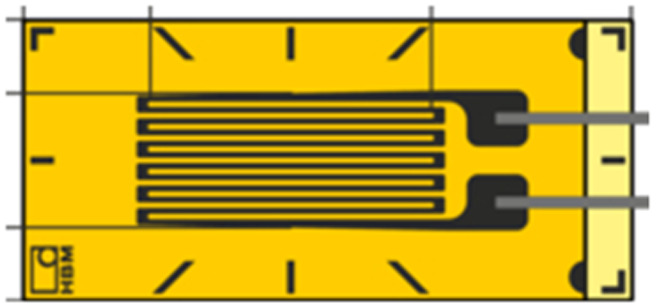	https://www.hbm.com/en/4561/ly-linear-strain-gauges-with-1-measurement-grid/
Techni Measure	8.8 × 3.5 mm	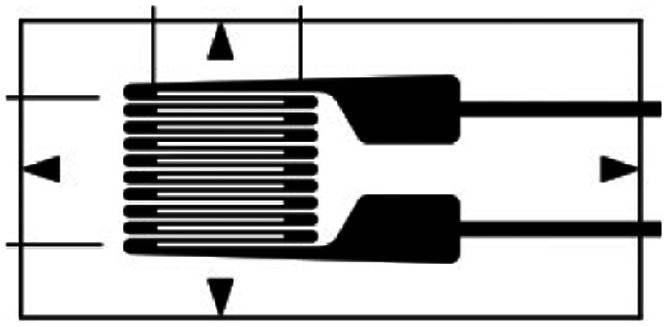	https://store.technimeasure.co.uk/product/fla-3-11-3lt/

### 3.2 SoC monitoring

**Fig. 4:**
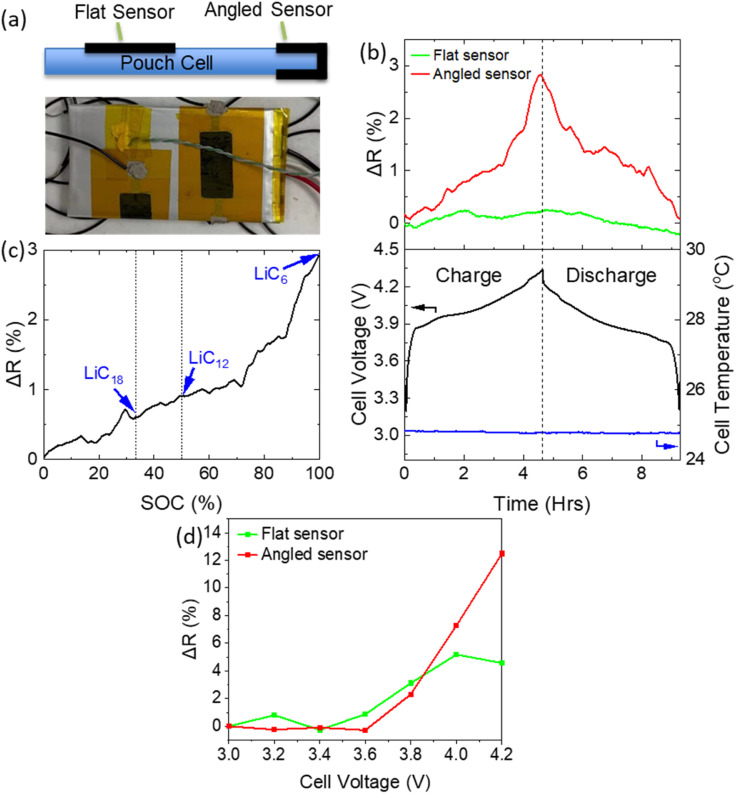
Galvanostatic testing of pouch cell. (a) Schematic and photograph of “flat” and “Angled” sensors mounted on a pouch cell. (b) Cell voltage and sensor resistances during two charge–discharge cycles at 0.2 C. (c) Plot showing the change in sensor resistance (Δ*R*) as s function of cell SoC. (d) Evolution in sensor resistance during potentiostatic cycling of pouch cell.

A commercially available pouch cell (Varta LPP 503562) was utilised to assess the ability of the sensor to measure SOC. Two graphene sensors were mounted on the cell in two different orientations to optimise placement for detection of volumetric change. The first was laid flat on top of the cell (“Flat sensor”) while a bending profile was introduced to the second sensor, as it was angled around the side of the cell (“Angled sensor”) as shown in Fig. 4a. The cell was then charged and discharged at 0.2 C in a temperature-controlled environment at 25 °C. Both ambient and cell temperature were monitored throughout and showed little change. The cycling current of 0.2 C was chosen so as to achieve full lithiation of the graphite, while minimising any temperature fluctuations (enabling isolation of volumetric changes due to lithiation alone). Fig. 4b shows the variation in cell voltage and sensor resistance. A clear correlation between resistance and cell voltage is visible for both sensors. However, the sensitivity of the sensor was highly dependent on its mounting profile. The angled sensor demonstrated a resistance change amplitude (Δ*R*) of 105 Ω (2.88% of initial), compared with just 7 Ω (0.3% of initial) in the case of the flat sensor. This clearly indicates that the introduction of a bending profile to the sensor results in a higher sensitivity and that the choice of mounting position is critical for the extraction of useful data. The importance of the mounting position has been observed previously,^17^ and can be attributed to a variation in cell expansion with location (specifically a greater level of strain experienced by the angled sensor). The pouch cell responded to internal stress through a dramatic expansion in the *z*-direction, and a much smaller expansion in the *x*–*y* direction (in the plane of the pouch cell). While the flat sensor only experienced strain associated with cell expansion in the *x*–*y*, the angled sensor experienced strain due to expansion in both the *x*–*y* and *z* directions. The bending profile was crucial in detecting this expansion mode. The high level of sensitivity was maintained throughout multiple charge–discharge cycles (Fig. S2†), whereby the sensor resistance closely tracked the charge/discharge profile. Fig. 4c shows a plot of Δ*R vs.* cell SoC, demonstrating the usefulness of this technique for SoC monitoring. A non-linear curve (with more rapid expansion at higher SoC) is expected from computational and XRD analysis of the lithiation of graphite.^12^ Specifically, the lithiation process is typically divided into several stages: LiC_18_, LiC_12_ & finally LiC_6_, associated with volume expansions of 3.6%, 4.6% and 10% respectively.

To extract the relationship between cell expansion and voltage, potentiostatic charging was performed on a similar pouch cell. Successive voltage steps were applied to the cell in the range 3.0–4.2 V, in increments of 0.2 V, and the potential was applied for a stabilisation period of 1 hour to allow the cell to reach a steady state, at which points values for Δ*R* were measured. Fig. 4d shows the variation of sensor resistance with cell voltage throughout the charging process. Again, both sensors showed an increase in resistance with increasing voltage. The sensors show little change in the range 3.0–3.6 V, but significant increases at potentials above this range. This is consistent with the voltage profile of the NMC‖graphite chemistry, in which the majority of cell capacity lies above 3.6 V.^24^ This indicates the potential of these sensors to also monitor cell open circuit voltage. Notably, a greater level of sensitivity was exhibited during the potentiostatic charge, whereby the sensor underwent a 12% increase in resistance compared with just 3% during the galvanostatic charge phase. This can be attributed to the higher SoC reached by charging potentiostatically, as the cell was placed at 4.2 V for 1 hour which enabled full lithiation of graphite. The expansion associated with lithiation of graphite is weighted towards high levels of charge. Some variation in the response from sensor to sensor was also observed, which is expected to be eliminated through more automated production and mounting methods.

Although they are not studied in this report (as we focus on commercially available cells), cells containing next-generation alloy-based anode materials (like Si or Sn) are expected to exhibit far greater dimensional changes during normal charge and discharge (Si expands by a factor of 400% during lithiation^25^). Such increased levels of expansion would be expected to enhance the accuracy of SoC determination by these sensors. Furthermore, this expansion is often associated with cell degradation (material pulverization and delamination),^26^ and expansion monitoring is therefore a key enabler for these next-gen cells.

### 3.3 SOH & safety monitoring

In addition to information on cell SOC, detection of volume expansion can provide valuable insights into cell SOH. In particular, common degradation processes of LIBs such as SEI buildup or gas generation can result in irreversible expansion of the cell.^13^ Early identification of the occurrence of these processes could allow mitigation steps to be taken, prolonging the lifetime of the cell and wider system. Furthermore, identification of specific non-functioning cells within a battery pack would allow the BMS to account for this, avoiding total pack failure.

#### 3.3.1 Irreversible expansion during normal operation

**Fig. 5:**
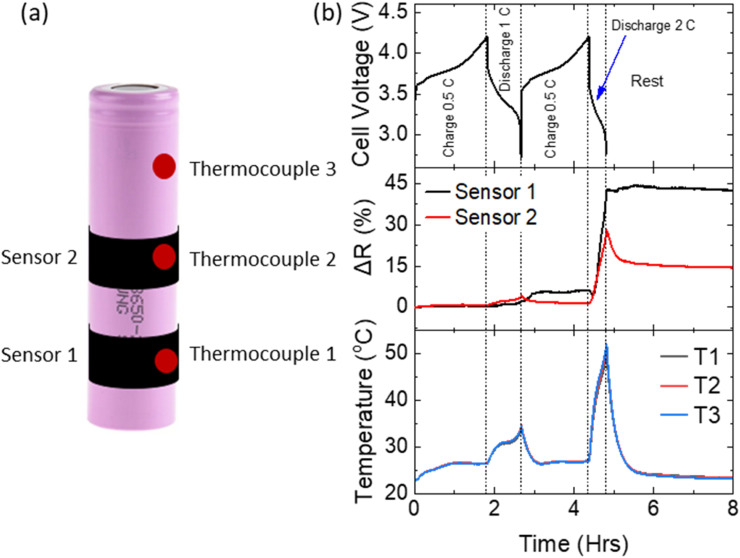
(a) Schematic showing mounting positions of two sensors and three thermocouples on an 18 650 cylindrical cell. (b) Cell voltage, % change in resistance and cell temperature as a function of time during charge and discharge.

Two sensors were mounted on a cylindrical cell (ICR18650-26J), as shown in Fig. 5a. Three K-type thermocouples were also attached, two were placed at the same height as the sensing elements, and the third was placed close to the top (positive) terminal of the cell. The cell was cycled by charging at 0.5 C, discharging at 1 C, charging at 0.5 C, and finally discharging at 2 C (2 C was the maximum discharge rate recommended by the manufacturer). As shown in Fig. 5b, the thermocouples measured a significant rise in cell temperature, particularly during discharge at 2 C, which was constant along the length of the cell. Both graphene sensors showed significant changes of resistance during the 2 C discharge steps, with sensor 1 exhibiting a much stronger response during the 2 C discharge. This response can be attributed to a combination of sensor temperature increase and volumetric changes in the cell itself due to the high temperature. Since the temperature was identical at all locations, the higher response of sensor 1 indicates a greater cell expansion near the negative terminal. This observation is consistent with previous observations of variations in cylindrical cell expansion across its length (associated with gradients in cell current and negative electrode potential).^19^ After the 2 C discharge, the cell was placed at open circuit and allowed to return to ambient temperature. Some reversibility in the resistance of sensor 2 was observed, whereas the resistance of sensor 1 did not revert at all. This indicates that even a relatively low discharge current of 2 C (within the specification range of the cell) resulted in a permanent deformation of the battery casing, and that the non-uniformity across the length of the cell could be detected through the mounting of multiple sensors. Such deformation can lead to ageing and deterioration of the cell SOH.

#### 3.3.2 Abuse testing

To further assess the performance of the graphene sensors in evaluating cell SOH, a pouch cell was subjected to abuse testing designed to simulate common cell failure modes, and to accelerate the aging process. The “overcharge” test (as specified in IEC62660-2) is designed to simulate a situation in which a cell is unintentionally charged beyond its rated charging voltage. This failure mode is common in battery packs as the BMS does not typically measure and control cell voltage individually, but rather total pack voltage. This situation is tolerable with identical pristine cells at 100% SOH, however cells will often degrade at varying rates, leading to mismatches and the accidental overcharging of certain cells.^1^

Charging the cell beyond its rated voltage will induce several undesirable electrochemical reactions. Firstly, the overdelithiation of the cathode will result in metal dissolution and a release of oxygen.^27^ Secondly, the overlithiation of the anode causes increased film thickness and rapid increase in temperature due to lithium plating.^28^ Thirdly, the electrolyte will react at the cathode solid electrolyte interface, decomposing and generating a large amount of heat.^29^

**Fig. 6:**
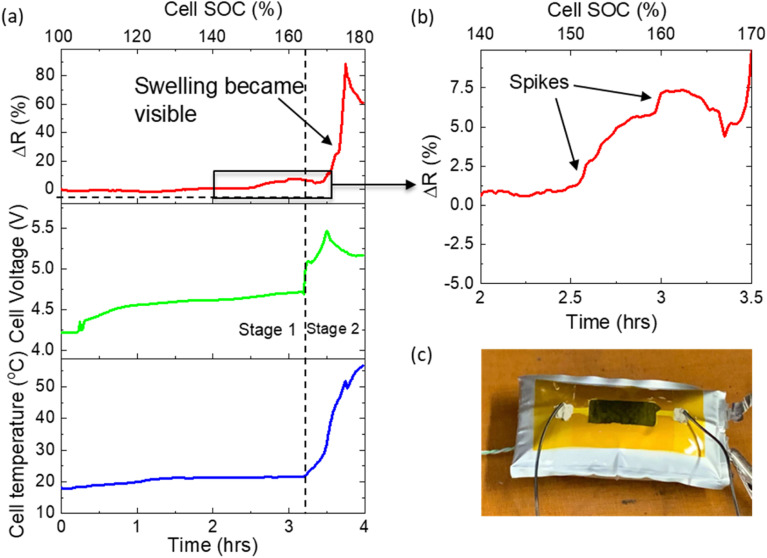
Overcharge test of pouch cell. (a) Evolution of sensor resistance, cell voltage and cell temperature during overcharge test. (b) Higher magnification of sensor resistance in region in which resistance spikes are visible. (c) Photo of swollen pouch cell with mounted sensor after test completion.

Here, a pouch cell (with mounted flat sensor) was fully charged to 4.2 V, after which a further charging current of 0.2 C was applied until the point of cell failure. Fig. 6a shows the evolution of sensor resistance, cell voltage and cell temperature throughout the test. A gradual increase in cell voltage to 4.7 V was observed (∼3.25 hours, stage 1), after which it rapidly increased to 5.5 V (stage 2). The first stage can be attributed to complete delithiation of the cathode. Initially this lithium will be accommodated in the small anode capacity excess (10–15%),^30^ typically designed into LIBs to avoid Li plating. Further Li will be consumed in SEI formation and finally, Li plating on the anode surface. At the end of stage 1, there was insufficient Li in the cathode to maintain the charging current, resulting in the onset of electrolyte decomposition, with associated large increase in cell voltage and temperature (stage 2). Little change in cell temperature or cell appearance was observed in the first stage. Physical swelling of the cell due to gas generation became visible to the naked eye during the second stage at approx. 3.5 hours. Concurrently, the resistance of the sensor increased dramatically by several kΩs.

Fig. 6b shows a high magnification of the sensor resistance during the latter part of stage 1. Interestingly, several strong spikes were observed, during which resistance changed rapidly (approx. 100 Ω min^−1^, far more rapidly and to a greater magnitude than that observed during standard cycling). At this point in the test, no significant changes to cell voltage, temperature or visual appearance had occurred. This volume expansion may be attributed to the release of oxygen from the cathode material and a change in anode thickness due to Li plating causing pressure build-up inside the cell. This pressure was equalized through small expansion/swelling events, not visible to the naked eye, but detected by the sensor. The final large peak in stage 2 can be attributed to further gas generation. The presence of these early rapid volume expansions (and their detection by the sensor) indicates that the monitoring of cell volume expansion can detect poor cell SOH, providing advance warning of impending extreme overcharge, and thus enable the BMS to prevent propagation of cell failure to the entire system.

## 4 Conclusion

A new resistance strain gauge designed to monitor LIB cell volume changes was assessed. The sensor, based on a graphene thin film percolative network, demonstrated strong sensitivity to the small volume changes observed during normal charging and discharging of both pouch and cylindrical cells. The mounting position of the sensor on the cell was crucial for this detection, with a sensor mounted to introduce a bending profile demonstrating an order of magnitude increase in sensitivity compared with a similar sensor mounted in a flat orientation. The recording of sensor resistance during standard charge–discharge cycling enabled a relationship with cell SoC to be extracted, meaning that the sensor could be used to determine SoC without interruption to cell operation. The sensor was also assessed for its ability to detect and quantify irreversible volume changes occurring due to cell degradation processes. Cell swelling due to gas generation during cell overcharge were correlated with a significant increase in sensor resistance. Crucially, the sensor detected smaller but significant volume changes prior to complete cell failure and can provide advance warning of such events. This advance warning can enable steps to be taken to prevent total cell and battery pack failure.

## Abbreviatons

EV: Electric vehicle
SoC: State of charge
SoH: State of health
BMS: Battery management system
NMC: LiNi_1−*x*−*y*_Mn_*x*_Co_*y*_O_2_
LFP: LiFePO_4_
R: Resistance
CID: Current interrupt device

## Conflicts of interest

There are no conflicts to declare.

## Supplementary Material

RA-013-D2RA07572E-s001
