# Supplementary material for: Monitoring state of charge and volume expansion in lithium-ion batteries: an approach using surface mounted thin-film graphene sensors
Source: RSC Adv. 2023 Mar 1;13(10):7045–54. doi: 10.1039/d2ra07572e (PMC9977397; doi:10.1039/d2ra07572e)
Supplement: RA-013-D2RA07572E-s001 [file RA-013-D2RA07572E-s001.pdf]

Electronic Supplementary Material (ESI) for RSC Advances.
This journal is © The Royal Society of Chemistry 2023

Monitoring state of charge and volume expansion in lithium-ion batteries: An approach using surface mounted thin-film graphene sensors

1 Supporting Information

Techniques used to monitor LIB volume changes				
Academic Reports				
Detection Method	Operational Principle	Notes	Observed volume change (%)	Ref
Resistance strain gauge	Change in electrical resistance of surface mounted element	Small footprint, inexpensive	0.3	[1–5]
Fibre Bragg gratings	Change in reflection of optical signal through surface-mounted fibre	Small footprint, expensive	0.015, 0.033	[6,7]
Dilatometry	Thickness gauge, typically single axis	Large footprint, expensive	2.1	[8–10]
Laser scanning	Measure of single-axis deformation	Large footprint, very expensive	2 - 2.8	[11,12]
Patents				
Title	Number	Assignee	Monitoring Method	Ref

Apparatus for predicting deformation of battery module	KR20180087041A	LG Chemical Ltd	Parallel Plate	[13]
Device for testing expansion of power battery cell and module	CN210346757U	Thornton New Energy Technology Changsha Co ltd	Parallel Plate	[14]
Arrangement and method for monitoring pressure within a battery cell	EP1856760B1	Philips	Strain Gauge	[15]
Strain measurement based battery testing	US9660299B2	Southwest Research Institute	Strain Gauge	[16]
Method for monitoring/managing electrochemical energy device by detecting intercalation stage changes	US9583796B2	Palo Alto Research Centre Inc.	Optical Sensor	[17]
Monitoring and management for energy storage devices	US9203122B2	Palo Alto Research Centre Inc.	Optical Sensor	[18]
Battery health state estimation based on swelling characteristics	US 2021/0197691 A1	University of Michigan	Pressure Sensor	[19]

Table S1: A comparison of reported techniques used to monitor LIB volume change in academic studies (upper table) and patents (lower table). This report addresses a novel form of resistance strain gauge. Typically, reported volume changes are smaller (< 0.5 %) for hard-cased cells, and larger (> 2 %) for soft-cased cells.

	Pouch Cell	Cylindrical Cell
	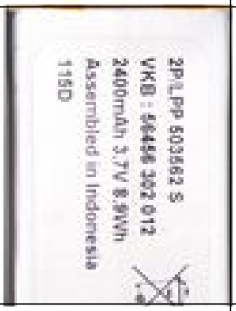	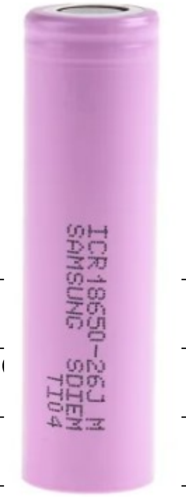
Manufacturer	Varta	[illegible]
Model	LPP 503562	[illegible]
Rated Capacity	1,160 mAh	[illegible]
Nominal Voltage	3.7 V	[illegible]
Upper cutoff voltage	4.2 V	4.2 V
Lower cutoff voltage	3.0 V	2.75 V
Standard charge/discharge current	2 A	1.3 A
Dimensions	64.5 x 35.5 x 5.3 mm	18 mm Ø x 65 mm

Table S2: Characteristics of commercially available battery cells used in this study. Parameters are taken from manufacturer’s datasheets

1.1 Influence of temperature on sensor output

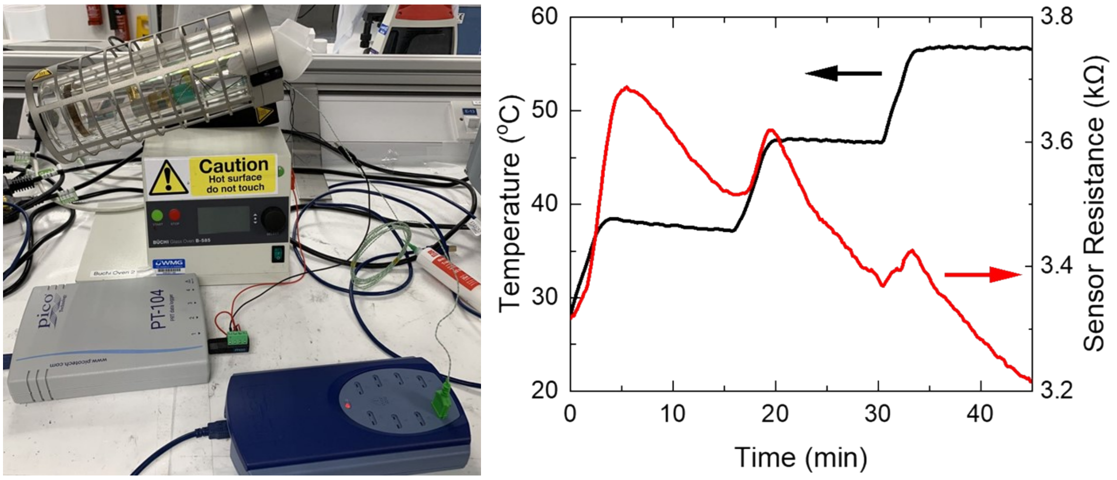

Figure S1: (a) Equipment setup used for determination of the influence of temperature, using oven (top of image) and resistance & temperature loggers (bottom of image). (b) Sensor resistance measured under temperature variation in the absence of significant physical strain.

The influence of temperature on sensor resistance (in the absence of strain) was assessed by mounting the sensor on a glass slide and placing in a temperature-controlled Buchi oven (see photograph in Figure S1). Glass was chosen as the substrate due to its relatively low thermal expansion co-efficient of approx. 10^{-5} K^{-1},[20] implying a volumetric expansion of just 0.03 % within this temperature range. We can therefore conclude that any change in resistance was brought about by temperature alone. The temperature of the oven was varied in discrete steps in the range 25 – 55 °C (this being the range in which batteries typically operate). The temperature dependence of conductivity for a sensor produced with ink GR024 is shown in Figure 3, which revealed complex behaviour. When the temperature was increased, the resistance initially increased i.e. there is a positive temperature coefficient as previously reported for printed graphene films.[21]. Notably, sensor resistance was not stable when the temperature was held at elevated values, displaying a tendency to revert to the original value. There is a strong time dependence of electrical conductivity on the relation processes in polymer-based systems, where the timescale of the polymer chains relaxation can affect the

time required to reach equilibrium. This can be attributed to the fact that the graphene percolative sensors are multi-phase materials which combine graphene particles with a complex polymer matrix. The thermal properties of such a multiphase material depend on the graphene material itself and on the properties of the polymers present; particularly on the presence of any phase transitions such as glass transitions or melting points which could affect the overall conductivity. As a result, the temperature dependence of conductivity for a particular graphene ink and sensors made from it, must be measured independently.

Any dependency of cell expansion or sensor gauge factor on temperature will need to be accounted for in the determination of cell SOC. Two solutions for this are possible, namely: (a) further development (choice of material, curing steps) of the polymer host material to reduce/eliminate the temperature-dependency of the sensor resistance, and (b) calibration to provide a quantitative determination of the temperature-resistance dependency, which can then be accounted for. Within the scope of this work, we focus on isolating and measuring the response of the sensor to SOC changes, and therefore temperature was held constant. Furthermore, during abuse testing, the sensor exhibited resistance changes so dramatic they could not have been related to temperature alone, and thus even in the absence of a full calibration, the sensor can provide valuable information on cell SOH.

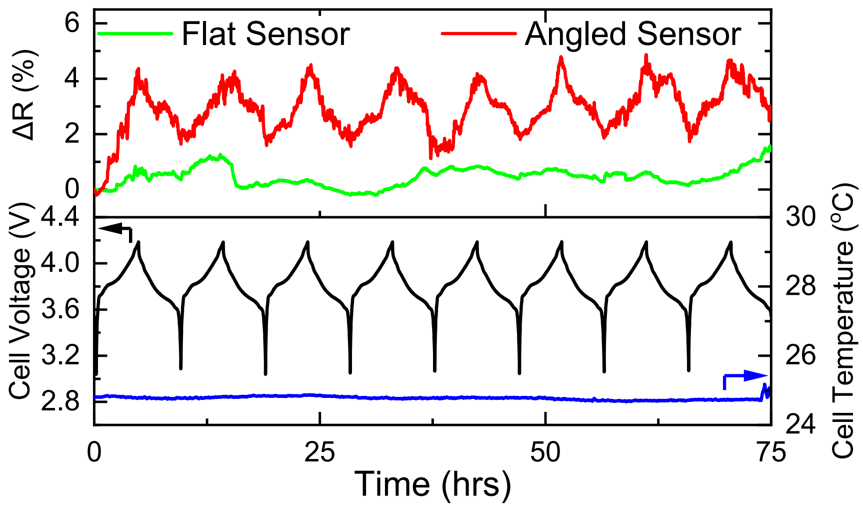

Figure S2: Change in flat and angled sensor resistances mounted on a pouch cell during repeated charge and discharge cycles. An initial irreversibility in sensor reading during cycle 1 was observed, whereby the sensor resistance did not return to its initial state. This was attributed to a combination of irreversible cell expansion caused by SEI formation during initial cycles (this was the first cycle the cell had undergone after presumed formation cycling at the point of manufacture), and an activation process in the sensor itself. It is our belief that a more thorough curing step for the sensors could eliminate initial irreversibility. In terms of the cell, since irreversible expansion is often associated with SEI formation, a more complete formation cycling stage would likely mitigate or fully eliminate this. Notably, when combined with OCV measurements, monitoring of irreversible cell expansion can provide useful information on cell SOH, as expansion can be well correlated with capacity loss.[22] During subsequent cycles, the resistance returns to a similar value at cell discharge.

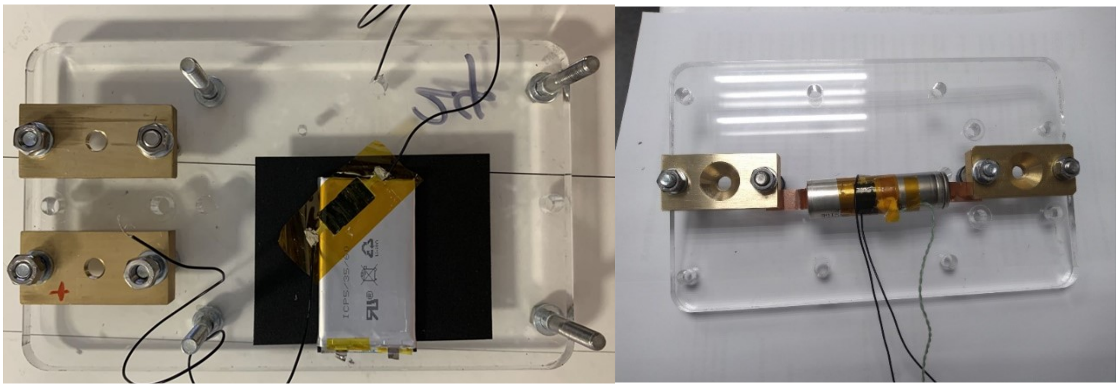

Figure S3: (a) Pouch and (b) Cylindrical cells in testing jigs with mounted sensor and thermocouple

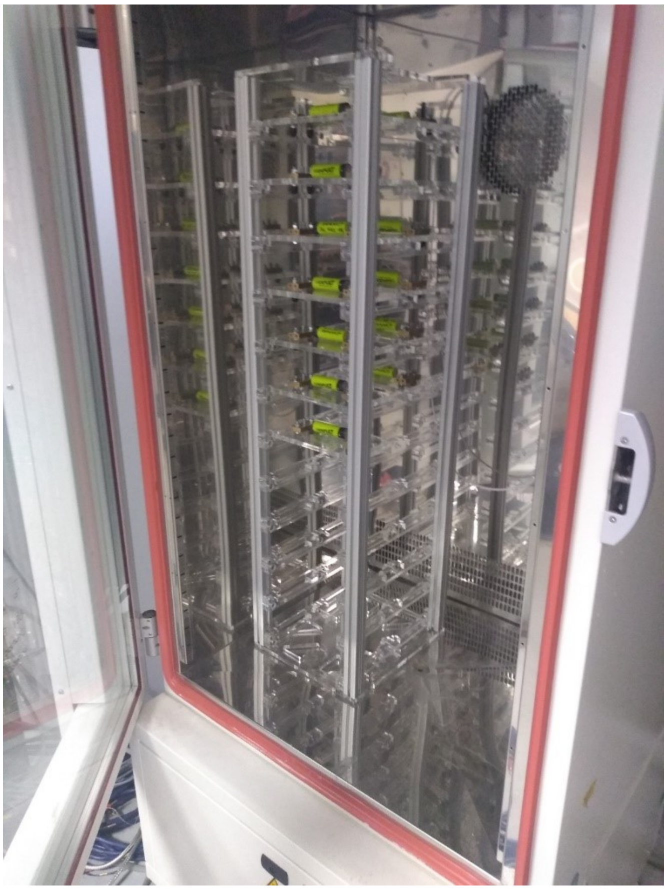

Figure S4: Thermal chamber including racks for cell testing

2 References

[1] L. Feng, S. Zhou, Y. Li, Y. Wang, Q. Zhao, C. Luo, G. Wang, K. Yan, *J. Energy Storage* **2018**, *16*, 84–92.

[2] M. Hempel, D. Nezich, J. Kong, M. Hofmann, *Nano Lett.* **2012**, *12*, 5714–5718.

[3] L. K. Willenberg, P. Dechent, G. Fuchs, D. U. Sauer, E. Figgemeier, *Sustain.* **2020**, *12*, DOI 10.3390/su12020557.

[4] R. Hickey, T. M. Jahns, *2019 IEEE Energy Convers. Congr. Expo. ECCE 2019* **2019**, 2460–2465.

[5] W. Ren, T. Zheng, C. Piao, D. E. Benson, X. Wang, H. Li, S. Lu, *J. Mater. Sci.* **2022**, *57*,

13560–13569.

[6] M. Nascimento, M. S. Ferreira, J. L. Pinto, *Batteries* **2018**, *4*, DOI 10.3390/batteries4020023.

[7] J. Meyer, A. Nedjalkov, A. Doering, M. Angelmahr, W. Schade, *Fiber Opt. Sensors Appl. XII* **2015**, *9480*, 94800Z.

[8] J. H. Lee, H. M. Lee, S. Ahn, *J. Power Sources* **2003**, *119–121*, 833–837.

[9] D. Bresser, J. Asenbauer, M. Kuenzel, T. Eisenmann, A. Birrozzi, J. K. Chang, S. Passerini, *J. Phys. Chem. Lett.* **2020**, *11*, 8238–8245.

[10] K. Y. Oh, J. B. Siegel, L. Secondo, S. U. Kim, N. A. Samad, J. Qin, D. Anderson, K. Garikipati, A. Knobloch, B. I. Epureanu, C. W. Monroe, A. Stefanopoulou, *J. Power Sources* **2014**, *267*, 197–202.

[11] Y. Zhao, F. B. Spingler, Y. Patel, G. J. Offer, A. Jossen, *J. Electrochem. Soc.* **2019**, *166*, A27–A34.

[12] B. Rieger, S. F. Schuster, S. V. Erhard, P. J. Osswald, A. Rheinfeld, C. Willmann, A. Jossen, *J. Energy Storage* **2016**, *8*, 1–5.

[13] K. I. M. D. YEON, K. J. I. N. HAK, C. Y. SEOK, *Apparatus for Predicting Deformation of Battery Module*, **2018**.

[14] L. I. ZEYA, X. ZHIJIANG, *Device for Testing Expansion of Power Battery Cell and Module*, **2020**.

[15] N. PETER, H. GEORG, R. KARL-RAGMAR, *ARRANGEMENT AND METHOD FOR MONITORING PRESSURE WITHIN A BATTERY CELL*, **2007**.

[16] X. U. J. QIANG, S. JOE, W. C. M, S. ROBERT, N. G. CHEUK, *Strain Measurement Based Battery Testing*, **2017**.

[17] S. BHASKAR, R. AJAY, K. PETER, S. L. WILKO, L. ALEXANDER, S. TOBIAS, S. S.

KUMAR, G. ANURAG, *Method for Monitoring/Managing Electrochemical Energy Device by Detecting Intercalation Stage Changes*, **2017**.

[18] A. G. Stefanopoulou, S. N. Abdul, Y. Kim, J. B. Siegel, *Monitoring and Management for Energy Storage Devices*, **2021**, US9203122B2.

[19] S. A. G, A. S. NASSIM, K. I. M. YOUNGKI, S. J. B, *State Of Battery Health Estimation Based On Swelling Characteristics*, **2021**.

[20] A. Makishima, J. D. Mackenzie, *J. Non. Cryst. Solids* **1976**, *22*, 305–313.

[21] I. Banerjee, T. Faris, Z. Stoeva, P. G. Harris, J. Chen, A. K. Sharma, A. K. Ray, *2D Mater.* **2017**, *4*, DOI 10.1088/2053-1583/aa50f0.

[22] P. Mohtat, S. Lee, J. B. Siegel, A. G. Stefanopoulou, *J. Electrochem. Soc.* **2021**, *168*, 100520.
